# First-line nivolumab plus ipilimumab for unresectable MPM in China: a cost-effectiveness analysis

**DOI:** 10.1186/s13023-023-02925-w

**Published:** 2023-10-16

**Authors:** Liu Yang, Xiaobing Song, Wanxian Zeng, Zhiwei Zheng, Wenqiang Lin

**Affiliations:** 1 Fudan University Shanghai Cancer Center (Xiamen), Xiamen, 361005 China; 2 Department of Pharmacy, Fujian Medical University Union Hospital, Fuzhou, 350100 China; 3 Department of Quality Management, Ganzhou Fifth People’s Hospital, Ganzhou, 341000 China; 4 The School of Pharmacy, Fujian Medical University, Fuzhou, 350122 China; 5 Department of Pharmacy, Cancer Hospital of Shantou University Medical College, Shantou, 515041 China

**Keywords:** Cost-effectiveness, Malignant pleural mesothelioma, Nivolumab, Ipilimumab, First-line treatment

## Abstract

**Background:**

The regimen of nivolumab plus ipilimumab (NI) has been recommended by the National Comprehensive Cancer Network Clinical Practice Guidelines in Oncology-Malignant Pleural Mesothelioma (Version 1.2022) and Chinese Guidelines for the Clinical Diagnosis and Treatment of Malignant Pleural Mesothelioma (2021 edition) as the first-line treatment for Malignant Pleural Mesothelioma (MPM). But whether immunotherapy has a financial advantage over conventional chemotherapy (pemetrexed plus cisplatin/carboplatin, C) is uncertain.

**Methods:**

Based on survival and safety data from the CheckMate 743 clinical trial (NCT02899299), a partitioned survival model was constructed using TreeAge Pro2022 software. The model cycle was set to 1 month and the study period was 10 years. The output indicators included total cost, quality-adjusted life year (QALY) and incremental cost-effectiveness ratio (ICER). One-way and probabilistic sensitivity analyses were used to assess the robustness of the results, considering only direct medical costs.

**Results and discussion:**

The ICER for group NI versus Group C was $375,656/QALY in all randomized patients, $327,943/QALY in patients with epithelioid histology, and $115,495/QALY in patients with non-epithelioid histology. The ICERs of all three different populations all exceeded the willingness-to-pay threshold (three times the per capita gross domestic product of China in 2021). The results of univariate sensitivity analysis showed that the price of pemetrexed and nivolumab had great influence on the analysis results. The results of the probabilistic sensitivity analysis show that the probability of the NI scheme being more economical in all three different populations was 0.

**What is new and conclusion:**

From the perspective of the Chinese healthcare system, in patients with unresectable MPM, NI has no economic advantage over C.

## Background

Mesothelioma is a rare tumor that arises from mesothelial cells in the pleura or elsewhere, of which approximately 81% originate from the pleura [1]. Malignant pleural mesothelioma (MPM) is a rare and fatal cancer with high invasiveness and a 5-year survival rate of only about 10% [2]. From 2004 to October 2020, platinum plus folic acid antimetabolizers (e.g., pemetrexed) were the only approved first-line treatment for MPM [3, 4]. However, the long-term survival outcome of chemotherapy remains poor [5–8]. In recent years, bevacizumab has been used to treat MPM, but its use varies by region [9]. A randomized, double-blind phase III trial (CheckMate 743) compared the safety and efficacy of first-line treatment for unresectable MPM with nivolumab plus ipilimumab (NI) or pemetrexed plus cisplatin/carboplatin (C). The results showed that NI significantly prolonged the median overall survival (OS) compared with C (14.1 months, 95% CI 12.4–16.3 months versus 18.1 months, 95% CI 16.8–21.0 months; Hazard ratio [HR] = 0.73, 95% CI 0.61–0.87), and 3-year OS rates (95% CI) were 15.4% (11.5–19.9) and 23.2% (18.4–28.2), respectively [10, 11]. The NI protocol has been recommended by the National Comprehensive Cancer Network Clinical Practice Guidelines in Oncology (NCCN Guidelines) for malignant pleural mesothelioma (version 1.2022) as the first-line treatment for MPM [12].

Although NI scheme has shown good safety and effectiveness, it is expensive. In particular, the price of ipilimumab in China is $77.96/mg [13]. The cost of the entire course of treatment (take the medicine every 6 weeks for about 6 months) is about $22,804, which is out of reach for many patients' families. According to the Guidelines for the Clinical Diagnosis and Treatment of Malignant Pleural Mesothelioma in China (2021 edition), nivolumab combined with ipilimumab is used for the first-line treatment of patients with unresectable MPM, amd no studies have evaluated the economics of this therapy. Our study aimed to evaluate the economics of NI versus C in the first-line treatment of unresectable MPM from the perspective of the Chinese healthcare system using pharmacoeconomic approaches.

## Methods

### Target population and procedures

The population included in this study is the same as that included in the clinical trial of CheckMate 743, that is, those who are 18 years old or older, who are histologically confirmed as unresectable MPM, who can't receive surgical treatment (with or without chemotherapy), and whose Eastern Cooperative Oncology Group (ECOG) performance status of 0 or 1 [10]. According to the CheckMate 743 clinical trial, nivolumab (3 mg/kg) was injected intravenously every 2 weeks and ipilimumab (1 mg/kg) was injected intravenously every 6 weeks in the NI group until the disease progressed, intolerant toxicity occurred or for up to 2 years. Patients in group C received intravenous injection of cisplatin (75 mg/m^2^) or carboplatin (area under concentration time curve: 5 mg/mL per min) combined with pemetrexed (500 mg/m^2^) every 3 weeks, with a maximum of 6 cycles [10].

### Model structure

**Fig. 1:**
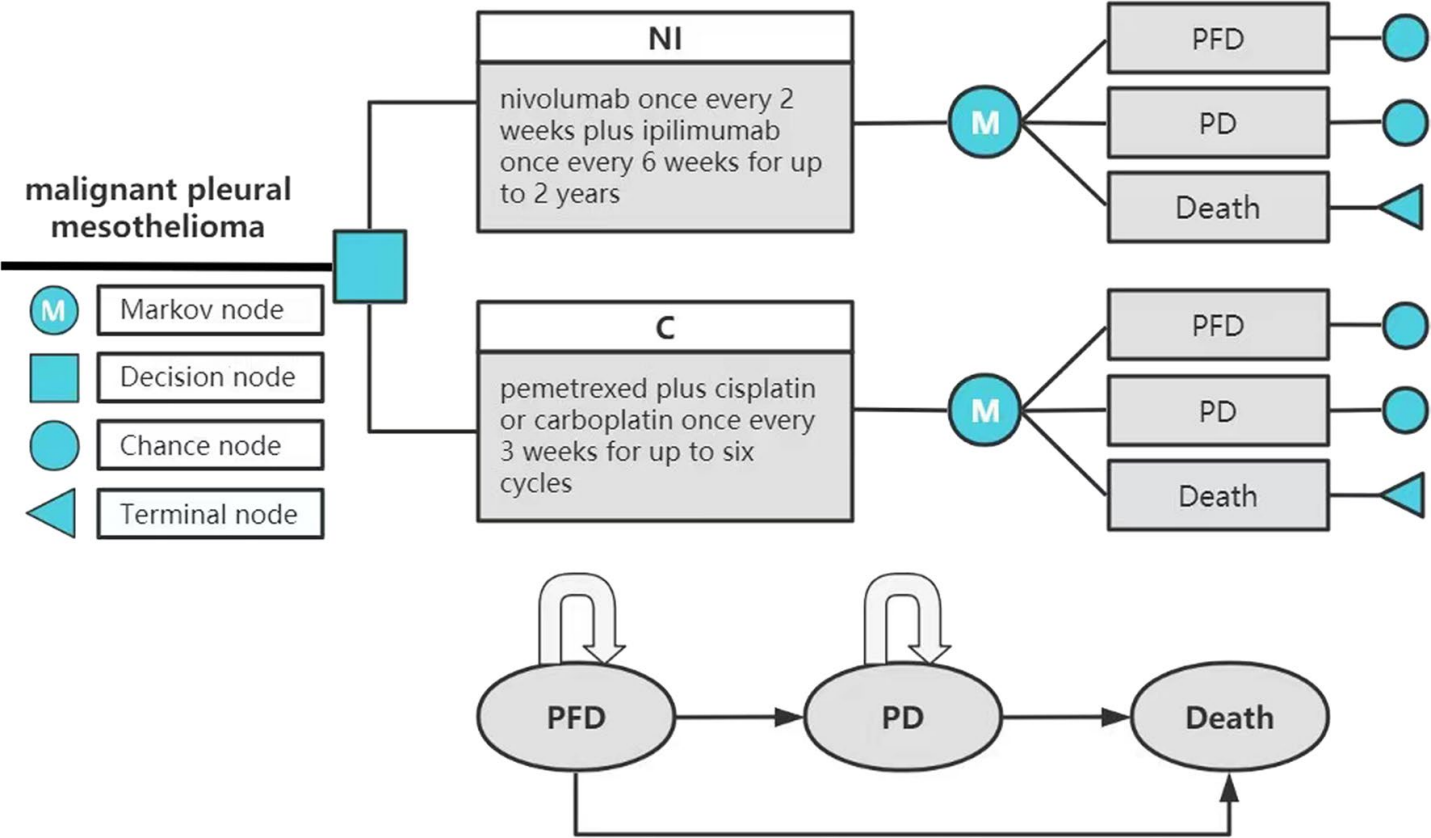
Partitioned survival model simulating outcomes for the CheckMate 743 trial. *NI* Nivolumab plus ipilimumab, *C* Pemetrexed plus cisplatin/carboplatin, *PFD* Progression-free disease, *PD* Progressed disease

The model was built by TreeAge Pro2022 software and analyzed statistically. The model includes three mutually exclusive health states: progression-free disease (PFD), progressive disease (PD) and death. It is assumed that all patients enter the model in PFD state and can maintain their designated health state or develop into another health state in each cycle (Fig. 1). And we assumed that the probability of PFD turning to death was equal to the natural mortality. According to related research, the 5-year survival rate of MPM patients was about 10%, so we set the running time of the model to 10 years. In order to facilitate the model operation and parameter calculation, we set the model period to 1 month. The main output results of the model include total cost, quality-adjusted life-years (QALY), and incremental cost-effectiveness ratio (ICER). And the cost and utility were discounted at a discount rate of 5%. Three times the per capita gross domestic product (GDP) of China in 2021 was used as the threshold of willingness to pay (WTP) [14]. In the original study, the author drew the Kaplan–Meier survival curve for the overall survival of all random population, epithelioid histology population and non-epithelioid histology population, so this study also constructed different Markov models for these three populations.

### Clinical data

Survival data for this study were obtained from the CheckMate 743 trial. In this study, GetData Graph Digitizer software was used to extract PFS and OS curve data from the CheckMate 743 study. Then, according to the method of Guyot et al. [15], R software was used to reconstruct the Kaplan–Meier survival curve and extrapolated to obtain a new survival curve. Fitted distribution functions include Weibull, log-logistic, log-normal, Gompertz, exponential, and gamma distribution functions. The detailed function formula of each distribution is shown in “Appendix 2 Fig. 11”. According to Akaike Information Criterion (AIC), Bayesian Information Criterion (BIC) and visual direct comparison of fitting curve and K-M curve to test the goodness of fit, the distribution function with lower AIC and BIC values and better visual simulation effect was selected as the fitting curve to obtain long-term clinical survival results. The AIC and BIC values of the fitting results of each function were shown in “Appendix 1 Table 3”.

In the CheckMate 743 study, the authors performed K–M curve analyses of progression-free survival (PFS) only for all randomized populations followed for at least 3 years (NI vs. C = 303 vs. 302). OS curves were plotted for all randomized population (NI vs. C = 303 vs. 302), epithelioid histological population (NI vs. C = 229 vs. 226), and non-epithelioid histological population (NI vs. C = 74 vs. 76). In this study, log-normal distribution and log-logistic distribution functions were used to fit the PFS curves of group NI and group C, and Weibull distribution, log-logistic distribution and exponential distribution functions were used to fit and extrapolate the OS curves of different populations in group NI. The OS curves of three different populations in group C were extrapolated by log-logistic distribution function. We performed internal model validation [16]. Which showed that the fitted PFS and OS curves closely matched those presented in clinical trials (“Appendix 2 Figs. 12, 13, 14, 15, 16, 17, 18, 19”). The model parameters and their value ranges were shown in Table 1.

**Table 1:** Model parameters

Variable	Baseline value	Range		References
		Minimum	Maximum	
NI: Log-normal PFS survival mode	λ = 1.93843, γ = 1.26135	–	–	[10]
C: Log-logistic PFS survival mode	λ = 7.53780, γ = 2.29427	–	–	[10]
NI: OS survival mode				
NI-A: WeibullPH OS survival mode	λ = 0.0241553, γ = 1.1284343	–	–	[10]
NI-E: Log-logistic OS survival mode	λ = 19.01452, γ = 1.51241	–	–	[10]
NI-N: Exponential OS survival mode	λ = 0.0412831	–	–	[10]
C: OS survival mode				
C-A: Log-logistic OS survival mode	λ = 14.25065, γ = 1.76236	–	–	[10]
C-E: Log-logistic OS survival mode	λ = 16.70245, γ = 1.70694	–	–	[10]
C-N: Log-logistic OS survival mode	λ = 9.03382, γ = 2.1$77.96 1	–	–	[10]
NI: Incidence of AEs, %				
Asthenia	0	–	–	[10]
Anemia	0.33	0.27	0.40	[10]
Neutropenia	0.67	0.53	0.80	[10]
C: Incidence of AEs, %				
Asthenia	4.20	3.36	5.04	[10]
Anemia	11.27	9.02	13.52	[10]
Neutropenia	15.14	12.11	18.17	[10]
Utility				
PFS	0.706	0.565	0.847	[19]
PD	0.565	0.452	0.678	[19]
Death	0	–	–	[19]
Asthenia	− 0.07	− 0.04	− 0.11	[20]
Anemia	− 0.073	− 0.037	− 0.110	[21]
Neutropenia	− 0.20	− 0.15	− 0.25	[21]
Drug cost per mg, USD				
Nivolumab	15.96	6.44	19.16	[13]
Ipilimumab	77.96	38.98	93.55	[13]
Pemetrexed	0.88	0.05	6.40	[13]
Cisplatin	0.12	0.01	0.83	[13]
Carboplatin	0.09	0.02	0.33	[13]
Vinorelbine	1.52	0.06	5.61	[13]
Gemcitabine	0.07	< 0.01	0.36	[13]
Administration IV, first hour, USD	7.83	6.27	9.40	[22]
Outpatient follow-up visit, per cycle, USD	69.13	51.85	86.29	[23]
AEs cost per 1-month cycle, first cycle only, USD				
Asthenia	96.05	67.24	124.87	[24]
Anemia	500.78	445.76	545.54	[21]
Neutropenia	434.57	0.00	1,290.65	[21]
Body area surface/m^2^	1.72	1.50	1.90	[18]
Weight/kg	65.00	48.75	81.25	[18]
Creatinine clearance/mL min^−1^	60	–	–	[18]
Discount rate	0.05	0	0.08	[25]

*NI* Nivolumab plus ipilimumab, *PFS* Progression-free survival, *C* Pemetrexed plus cisplatin/carboplatin, *OS* Overall survival, *A* All randomized patients, *E* Patients with epithelioid histology, *N* Patients with non-epithelioid histology, *AEs* Adverse events, *USD* United States Dollar, *PD* Progressed disease, *IV* Intravenous injection

### Cost and utility

Based on the perspective of the Chinese healthcare system, this study only considered direct medical costs, including drug costs, follow-up costs, drug injection costs, adverse event (AEs) treatment costs, and second-line treatment costs after progression. By comparing AEs in the NI and C groups, we included only three AEs with significant differences in incidence (asthenia [0% vs. 4.2%], anemia [0.3% vs. 11.3%], and neutropenia [0.7% vs. 15.1%]). According to the CheckMate 743 clinical trial, in which patients received first-line treatment with NI or C until disease progression, intolerable toxicity, or the maximum prescribed duration, the duration of first-line treatment in the NI group was considered to be 6 months (median = 5.6 months, IQR 2.0–11.4 months), and the duration of first-line treatment in group C was 4 months (median = 3.5 months, IQR 2.7–3.7 months). Furthermore, we assumed a probability of 0 to 1 for the use of cisplatin or carboplatin in group C. According to the NCCN Guidelines for MPM (version 1.2022) [12], pemetrexed (500 mg/m^2^) combined with cisplatin (75 mg/m^2^) or carboplatin (area under the concentration time curve of 5 mg/mL per min) was used as second-line therapy in the NI group, and all three drugs were given intravenously every 3 weeks and assumed the same probability range (0–1) for cisplatin and carboplatin. Group C received second-line nivolumab (3 mg/kg intravenously once every 2 weeks) with or without ipilimumab (1 mg/kg intravenously once every 6 weeks), vinorelbine (25 mg/m^2^ intravenously on days 1 and 8 every 3 weeks) or gemcitabine (1000 mg/m^2^ intravenously on days 1 and 8 every 3 weeks) [17]. The probability ranges for immunotherapy and chemotherapy were assumed to be equal, that is, the probability of using nivolumab alone plus the probability of using nivolumab plus ipilimumab was 0.5, and the probability of using vinorelbine plus the probability of using gemcitabine was 0.5, and the parameter P was set in the model as the probability of using immunotherapy in the second-line treatment of group C, and the probability range was set from 0 to 1. And at the same time, it was also assumed that nivolumab alone and nivolumab plus ipilimumab had the same probability range (0–1) for second-line immunotherapy in arm C, and the same probability range for vinorelbine and gemcitabine during chemotherapy (0–1). According to the NCCN guidelines and related references, it was assumed that the second-line platinum-based conventional chemotherapy in group NI would last up to 5 months, and the second-line immunotherapy in group C would last up to 3 months, and the mono-chemotherapy would last up to 16 months. The model assumed that all patients received second-line therapy after the initial progression of disease, and only drug costs and follow-up costs for second-line therapy were considered. Drug costs were derived from the median bid price of each province/municipality on Yaozh.com from 2017 to 2021 [13], and follow-up costs were obtained from published literature. In reference to the median age in the CheckMate 743 trial, the initial model patients had the following characteristics: age of 69 years, mean body weight of 65 kg, surface area of 1.72 m^2^, and creatinine clearance of 60 mL/min [18]. Other costs are shown in Table 1, and all costs are converted to US dollars at the exchange rate as of November 5, 2022 (RMB:USD = 7.1831:1).

The utility value represents the health-related quality of life for each health state. We assumed that AEs only happened in the first period. No outcome measures of health utility were addressed in the CheckMate 743 study, therefore, utility values and treatment costs for AEs in this study model were derived from other published literature. Since there was no accurate utility values of PFS and PD statuses in MPM patients before this, the utility values of PFS and PD statuses of patients in this study referred to the published utility values of non-small cell lung cancer, and we assumed that the utility value for the same health status were the same in both groups. The utility values for PFS status, PD status, and death status were 0.706, 0.565, and 0, respectively [19]. All utility values are shown in Table 1.

### Sensitivity analysis

A single factor sensitivity analysis was performed on the main parameters such as cost, utility and probability, and the results were presented in the form of a tornado plot. The basic value of drug prices adopts the median value of the winning bid prices of all provinces/municipalities in Yaozh.com from 2017 to 2021. The floating range was 50% downward and 20% upward from the basic value. The range of values for other parameters was determined based on the 95% confidence interval in the referenced literature or 20% above or below the base value, and the range of the discount rate was 0–8%. Probabilistic sensitivity analyses were performed by second-order Monte Carlo simulations to assess the overall robustness of the findings. A total of 1000 iterations were performed to calculate ICER values for each sampling of different treatment regimens, and the results were presented in the form of cost-effectiveness acceptability curves curves and scatter plots.

## Results

### Basic case analysis

The results of model operation showed that most patients died within 10 years, and the models of this study basically simulated the lifetime outcomes of MPM patients (the study period was 10 years). The basic analysis results were shown in Table 2. Among all random patients, compared with group C, the NI group could bring higher survival benefit (ΔQALY = 0.10), but also higher total cost (Δcost = $38,023). The ICER value was $375,656/QALY, which far exceeded the preset WTP threshold. In the population with epithelial histology, the NI regimen also had better survival benefit (ΔQALY = 0.12) and higher total cost (Δcost = $38,002) than the C regimen. The ICER value of the NI group compared with the C group was $327,943/QALY, which also exceeded the preset WTP threshold. In the non-epithelioid histological population, patients in the NI group had 0.33 QALYs more than those in the C group, with an incremental cost of $38,543 and an ICER value of $115,495/QALY, which was also much larger than the preset WTP threshold. It can be seen that in the three populations, although regimen NI can bring more survival benefits to patients compared with regimen C, the total cost also increases, and the ICER values are higher than 3 times of China's per capita GDP in 2021.

**Table 2:** Cost-effectiveness analysis

Strategies	Life years	Total costs ($)	QALYs	ICER, $/QALY
A				
NI	2.01	46,362	1.30	375,656
C	1.89	8339	1.20	
Incremental (NI vs. C)	0.12	38,023	0.10	
E				
NI	2.01	46,598	1.44	327,943
C	1.89	8596	1.33	
Incremental (NI vs. C)	0.12	38,002	0.12	
N				
NI	2.01	46,232	1.24	115,495
C	1.89	7689	0.90	
Incremental (NI vs. C)	0.12	38,543	0.33	

*QALY* Quality-adjusted life year, *ICER* Incremental cost-effectiveness ratio, *A* All randomized patients, *E* Patients with epithelioid histology, *N* Patients with non-epithelioid histology, *NI* Nivolumab plus ipilimumab, *C* Pemetrexed plus cisplatin/carboplatin

### Sensitivity analysis

**Fig. 2:**
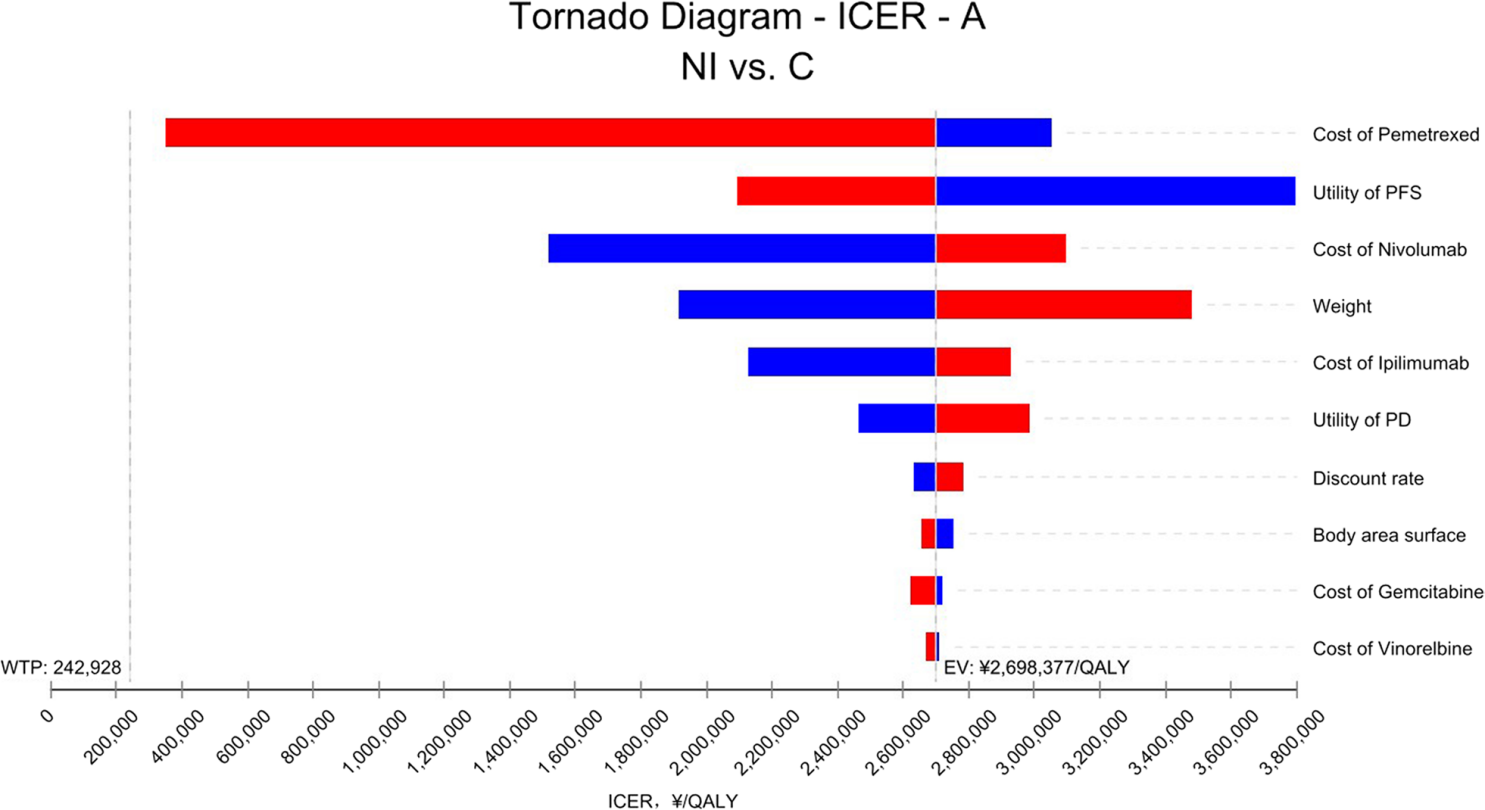
One-way sensitivity analysis in all randomized patients. *A* All randomized patients, *NI* Nivolumab plus ipilimumab, *C* Pemetrexed plus cisplatin/carboplatin, *PFS* Progression-free survival, *PD* Progressive disease, *WTP* Willingness to pay, *EV* Average value, *QALY* Quality-adjusted life year

**Fig. 3:**
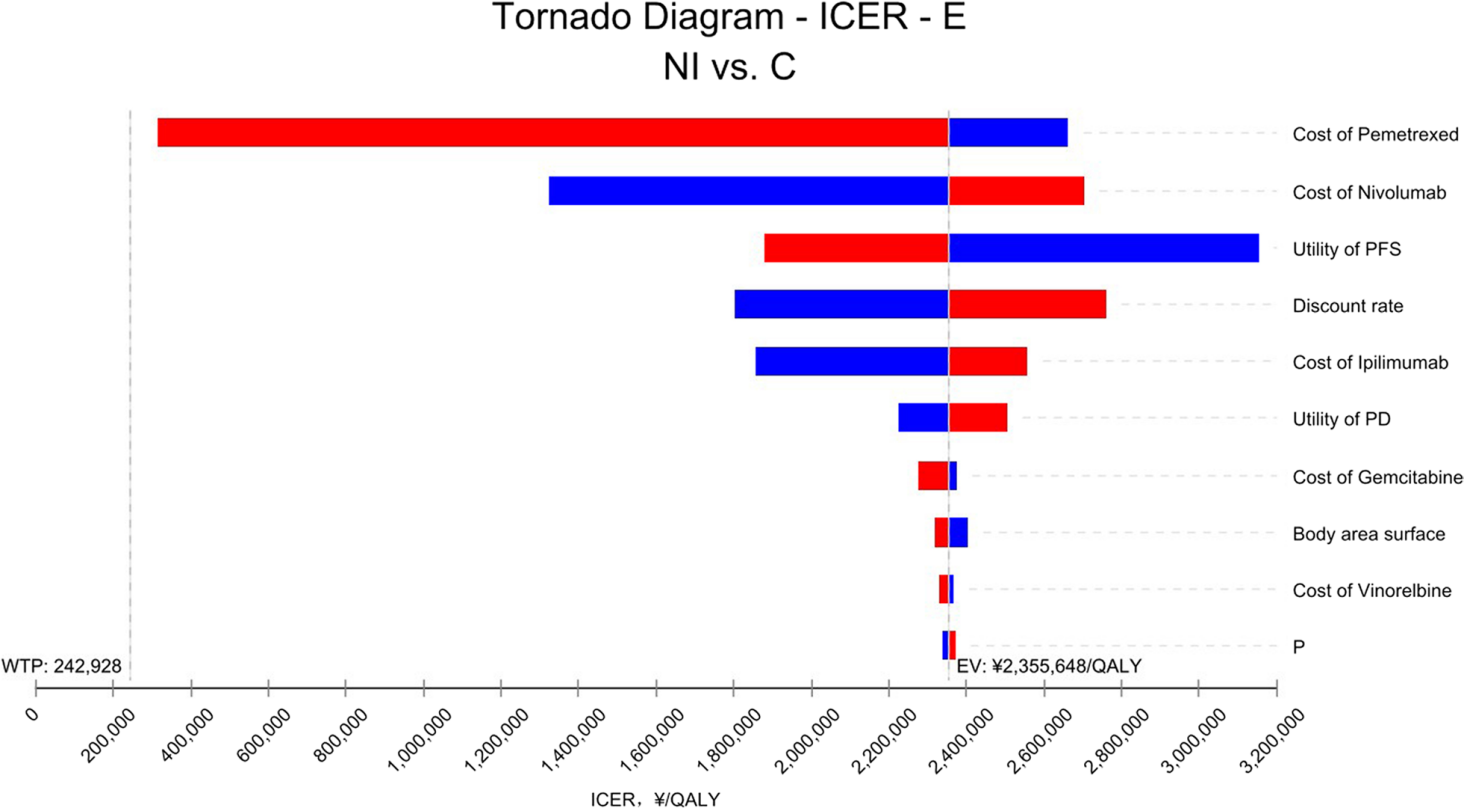
One-way sensitivity analysis in patients with epithelioid histology. *E* Patients with epithelioid histology, *NI* Nivolumab plus ipilimumab, *C* Pemetrexed plus cisplatin/carboplatin, *PFS* Progression-free survival, *PD* Progressive disease, *P* Probability of second-line immunotherapy in chemotherapy group, *WTP* Willingness to pay, *EV* Average value, *QALY* Quality-adjusted life year

**Fig. 4:**
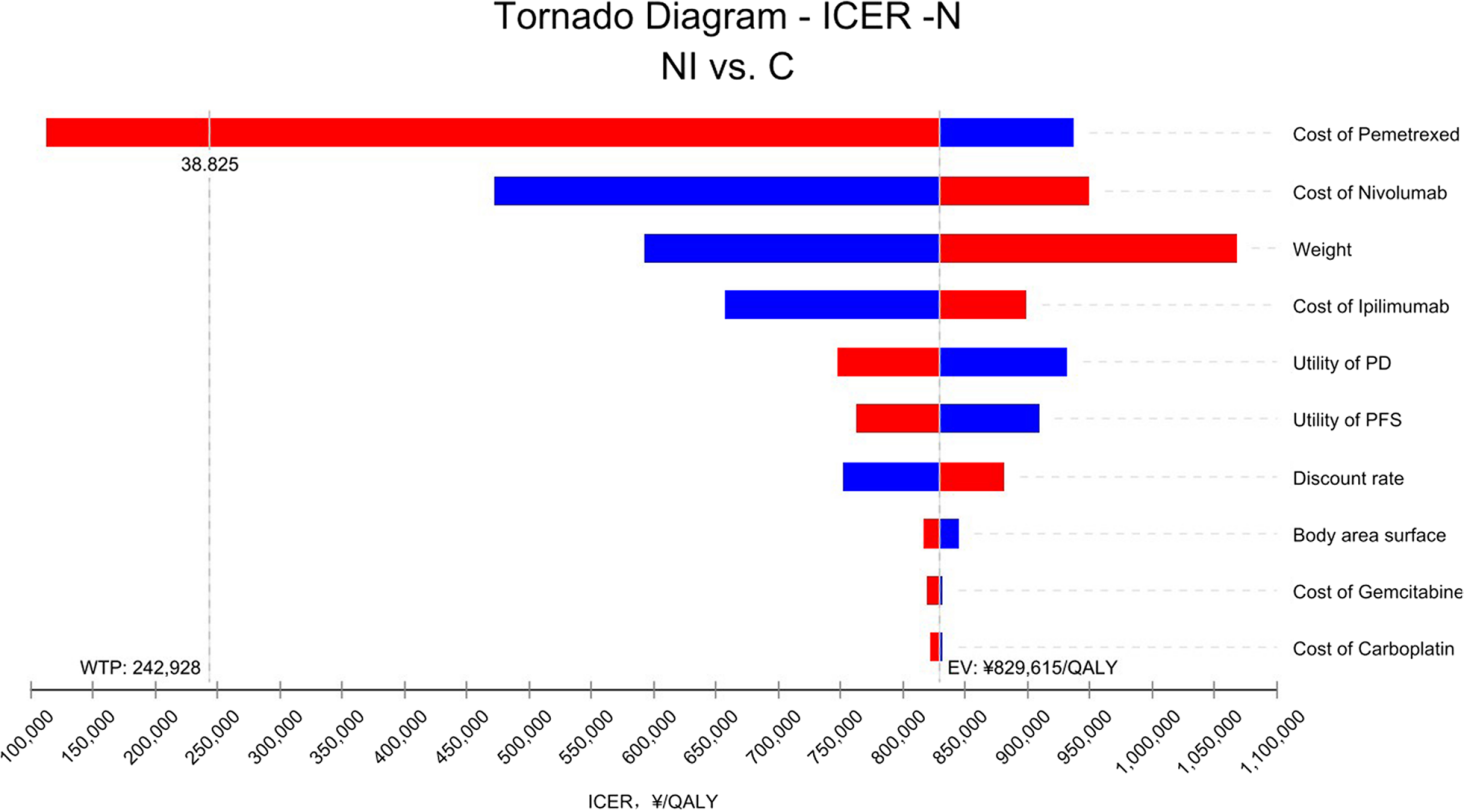
One-way sensitivity analysis in patients with non-epithelioid histology. *N* Patients with non-epithelioid histology, *NI* Nivolumab plus ipilimumab, *C* Pemetrexed plus cisplatin/carboplatin, *PD* Progressive disease, *PFS* Progression-free survival, *WTP* Willingness to pay, *EV* Average value, *QALY* Quality-adjusted life year

In all randomized and epithelioid histological populations, the price of pemetrexed, the price of nivolumab, and the utility value of PFS had a significant impact on the results. However, regardless of how these parameters were individually varied, the ICER value of the NI group compared with the C group was higher than the preset WTP threshold. The tornado diagram of one-way sensitivity analysis are shown in Figs. 2 and 3. In the non-epithelioid histological population, the price of pemetrexed, the price of nivolumab, and the weight of the patient had a significant impact on the results (Fig. 4). When the price of pemetrexed rose to $5.41/mg, the ICER value of group NI compared with group C was equal to 3 times GDP per capita, that is, when the price of pemetrexed was greater than $5.41, the ICER value of group NI compared with group C was less than 3 times GDP per capita, which was lower than the preset WTP threshold, which had cost-effectiveness advantages. However, no matter how other parameters were individually changed within the prescribed range, the ICER value of group NI compared with group C was higher than the preset WTP threshold, which did not have the cost-effectiveness advantage.

**Fig. 5:**
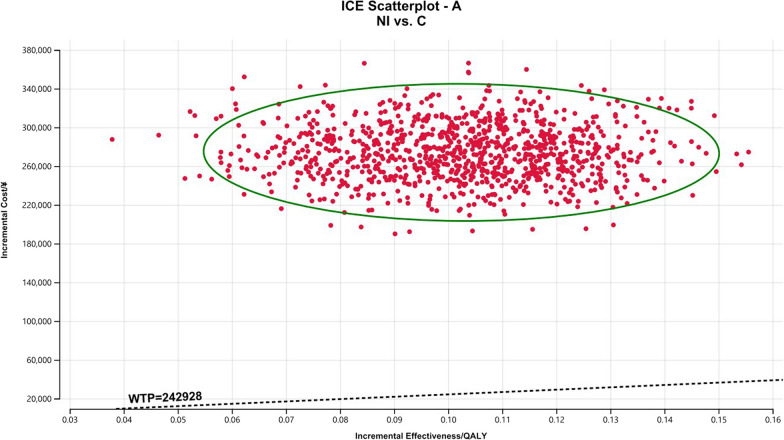
Scatterplot of probabilistic sensitivity analysis for all randomized patients. *A* All randomized patients, *NI* Nivolumab plus ipilimumab, *C* Pemetrexed plus cisplatin/carboplatin, *WTP* Willingness to pay, *QALY* Quality-adjusted life year

**Fig. 6:**
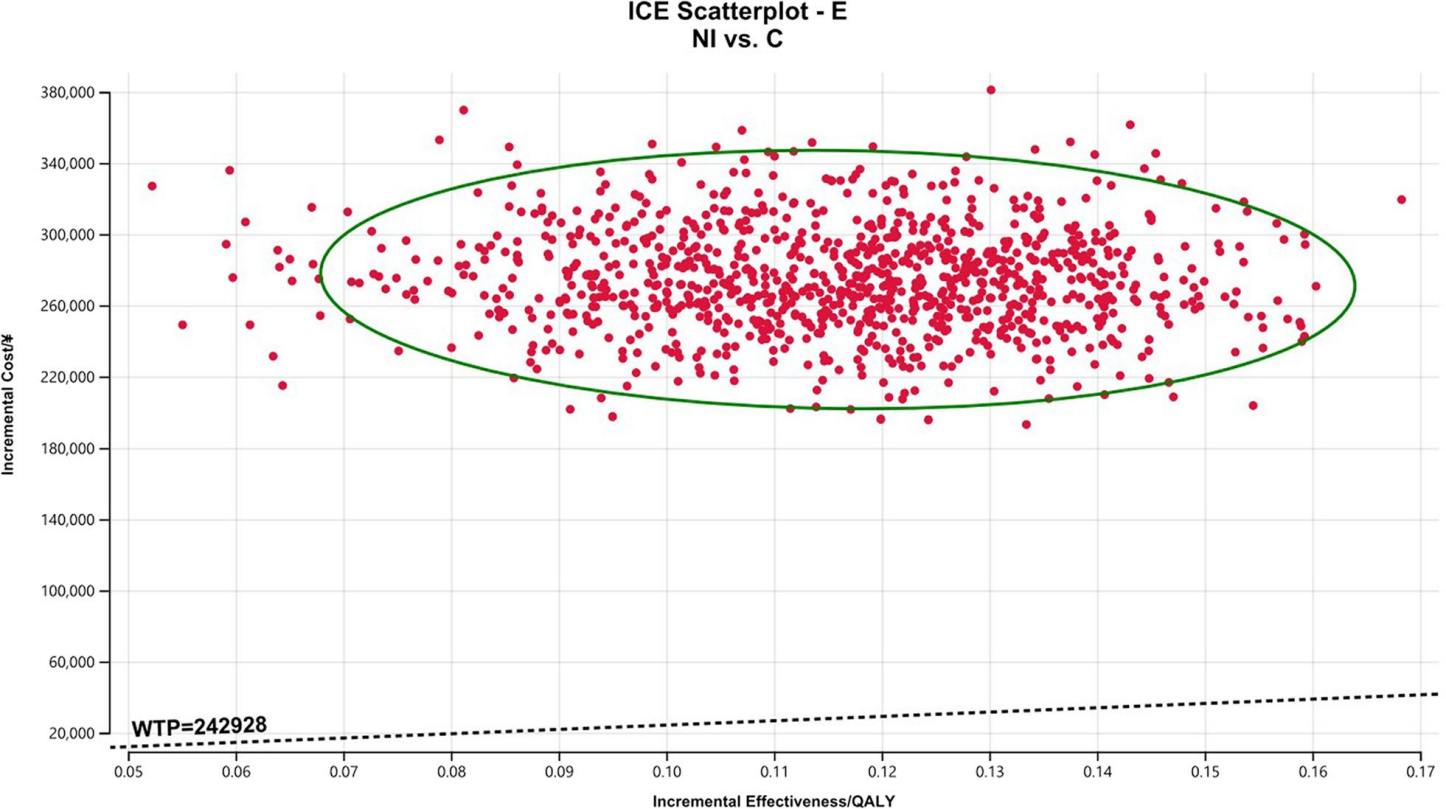
Scatterplot of probabilistic sensitivity analysis for all randomized patients. *E* Patients with epithelioid histology, *NI* Nivolumab plus ipilimumab, *C* Pemetrexed plus cisplatin/carboplatin, *WTP* Willingness to pay, *QALY* Quality-adjusted life year

**Fig. 7:**
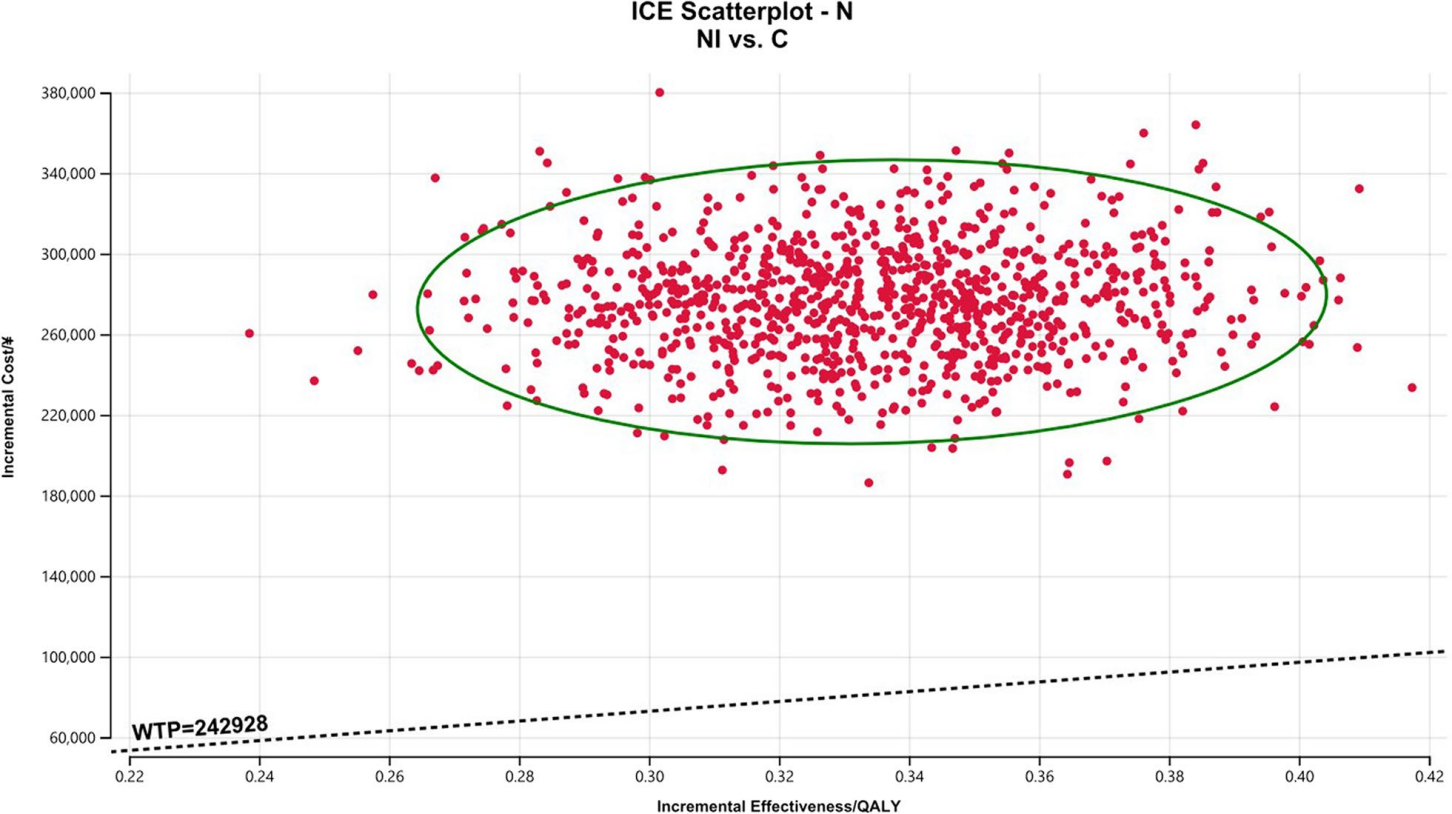
Scatterplot of probabilistic sensitivity analysis for all randomized patients. *N* Patients with non-epithelioid histology, *NI* Nivolumab plus ipilimumab, *C* Pemetrexed plus cisplatin/carboplatin, *WTP* Willingness to pay, *QALY* Quality-adjusted life year

**Fig. 8:**
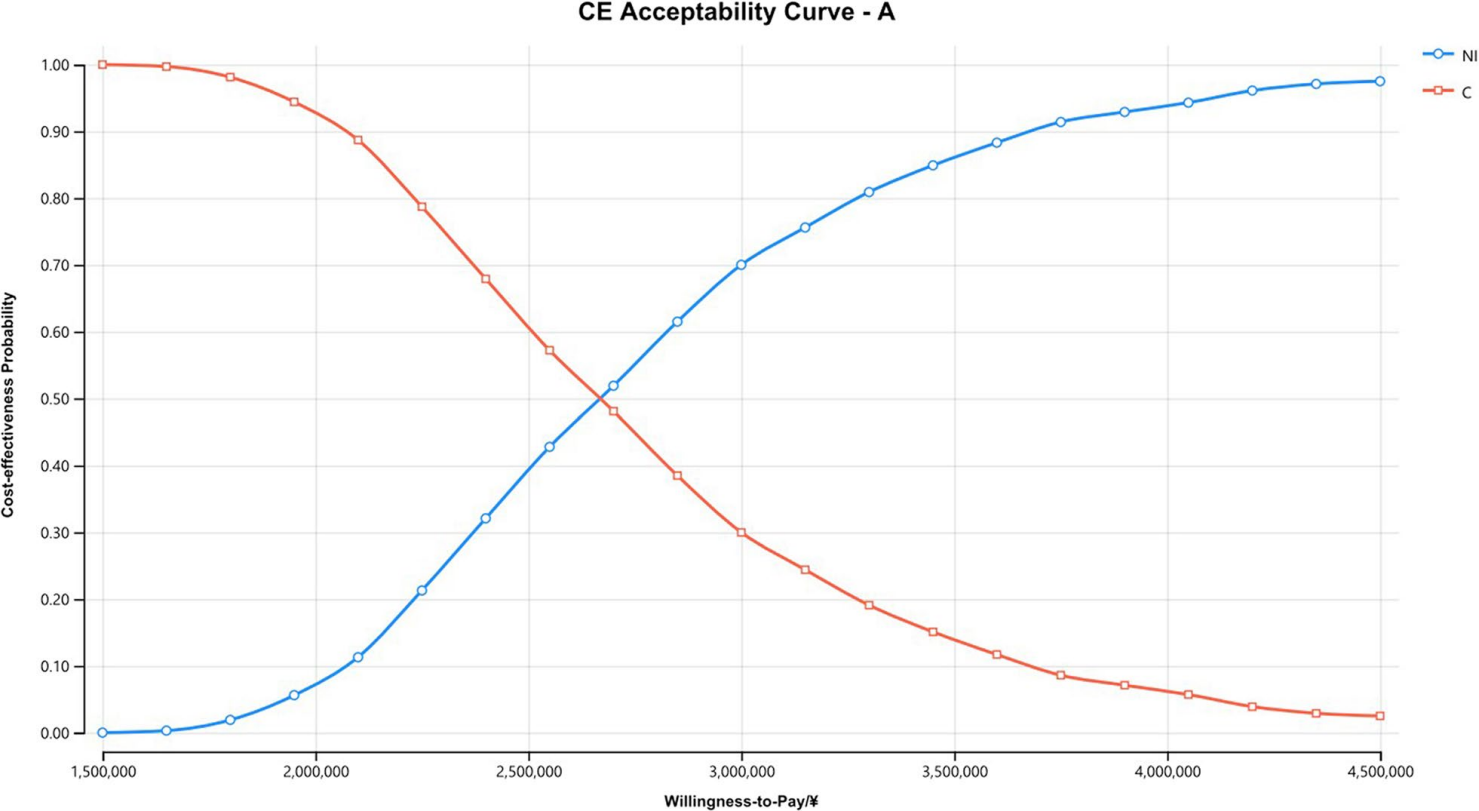
The cost-effectiveness acceptability curves of all randomized patients. *CE* Cost-effectiveness, *A* All randomized patients, *NI* Nivolumab plus ipilimumab, *C* Pemetrexed plus cisplatin/carboplatin

**Fig. 9:**
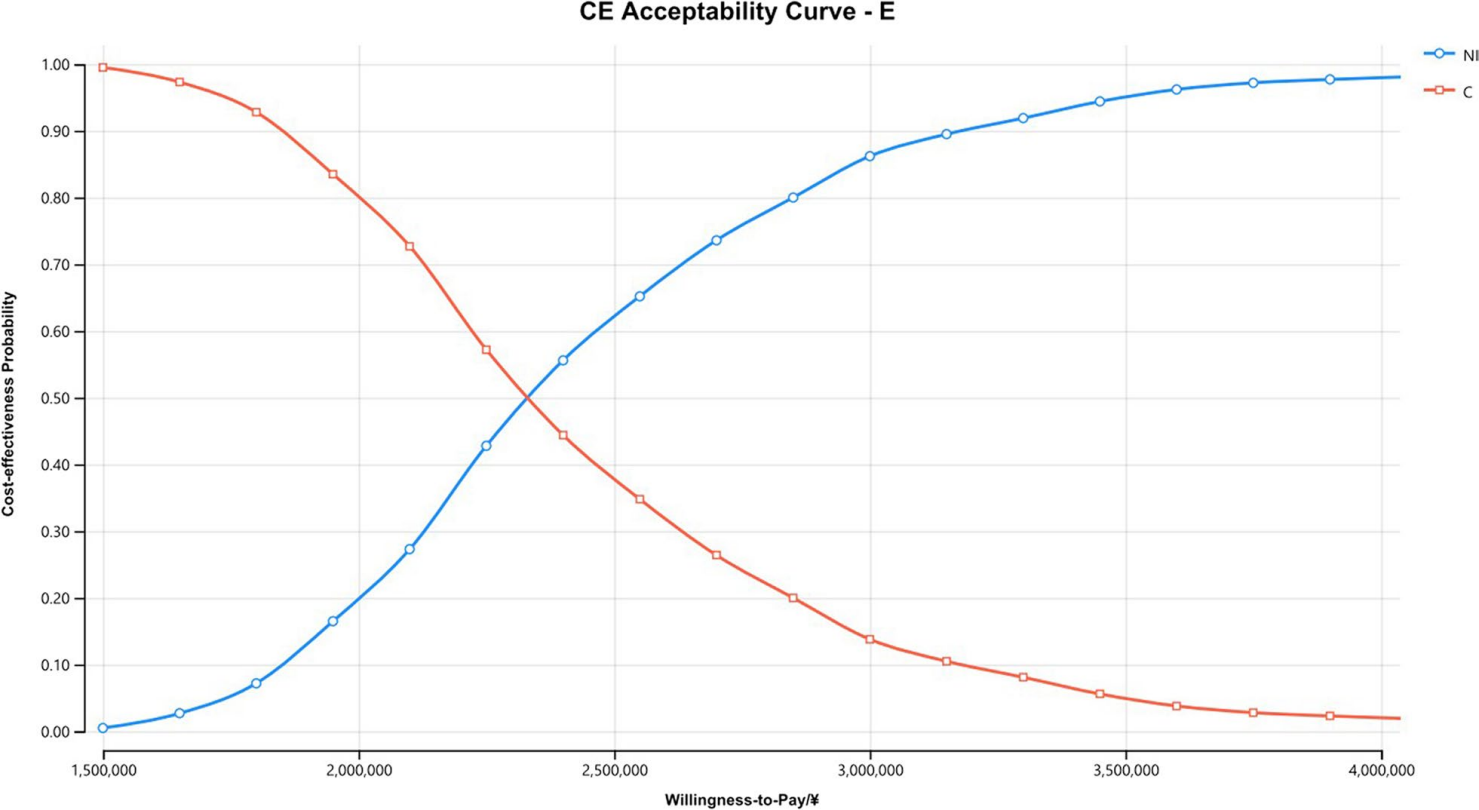
The cost-effectiveness acceptability curves of patients with epithelioid histology. *CE* Cost-effectiveness, *E* Patients with epithelioid histology, *NI* Nivolumab plus ipilimumab, *C* Pemetrexed plus cisplatin/carboplatin

**Fig. 10:**
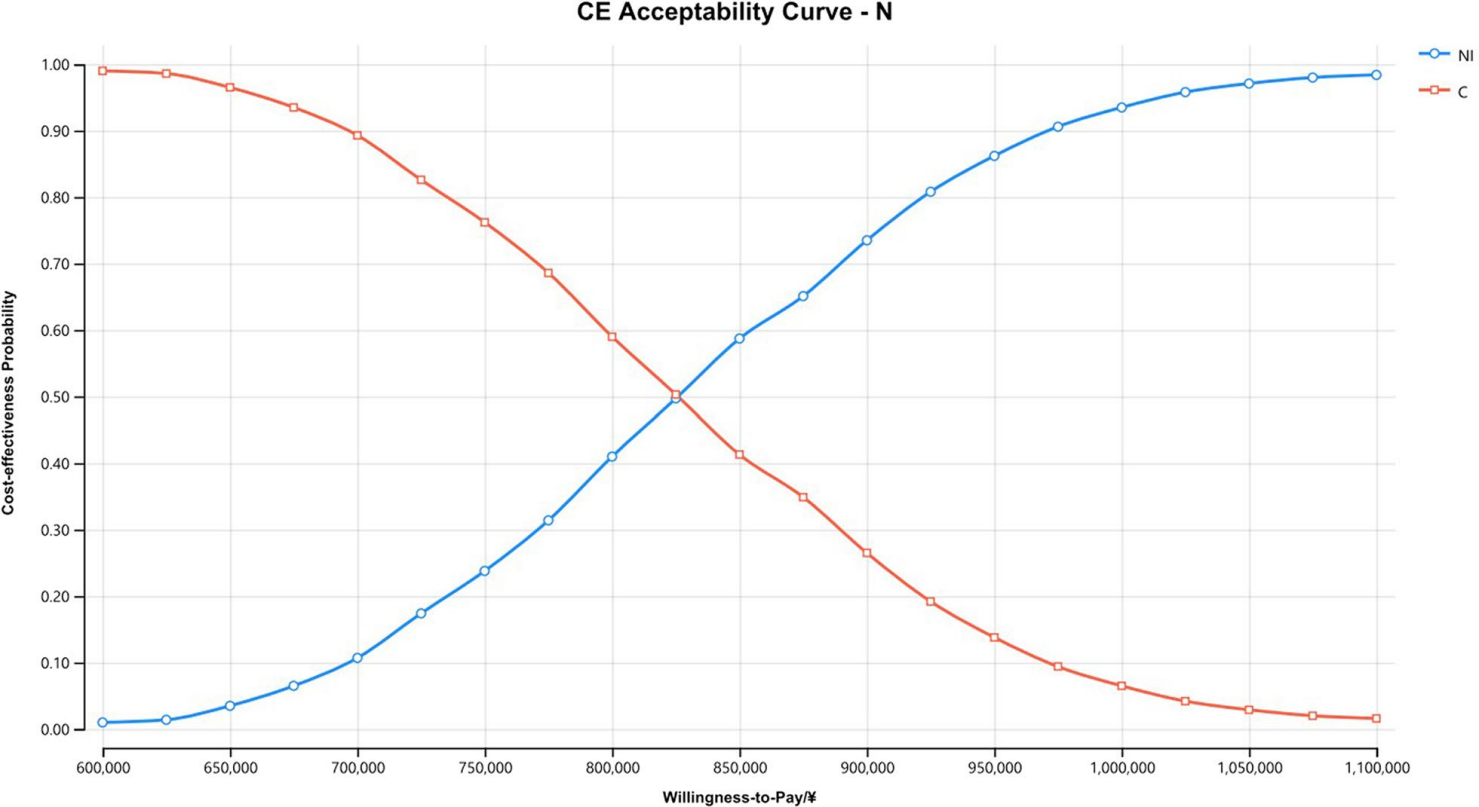
The cost-effectiveness acceptability curves of patients with non-epithelioid histology. *CE* Cost-effectiveness, *N* Patients with non-epithelioid histology, *NI* Nivolumab plus ipilimumab, *C* Pemetrexed plus cisplatin/carboplatin

**Fig. 11:**
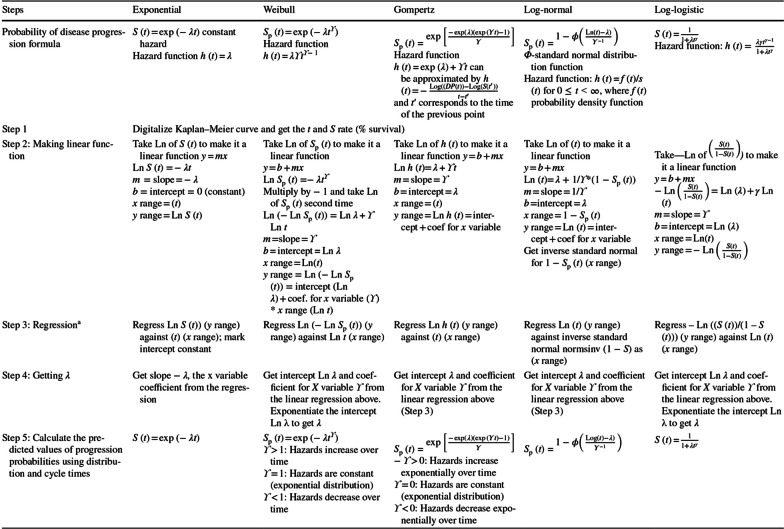
Key steps to fit commonly used parametric survival distributions to the non-parametric Kaplan–Meier curve

**Fig. 12:**
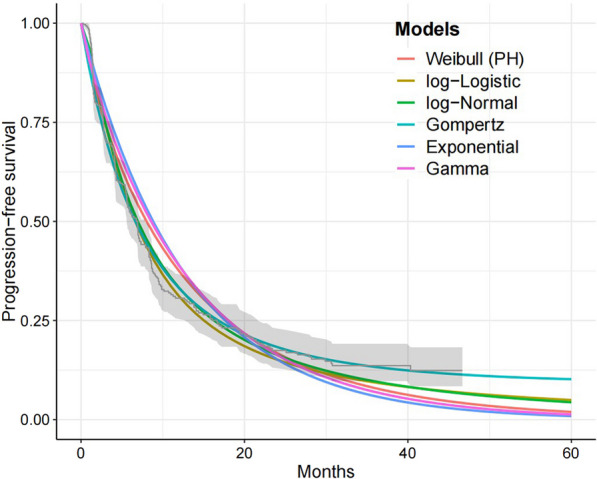
PFS(NI) derived from the model simulation

**Fig. 13:**
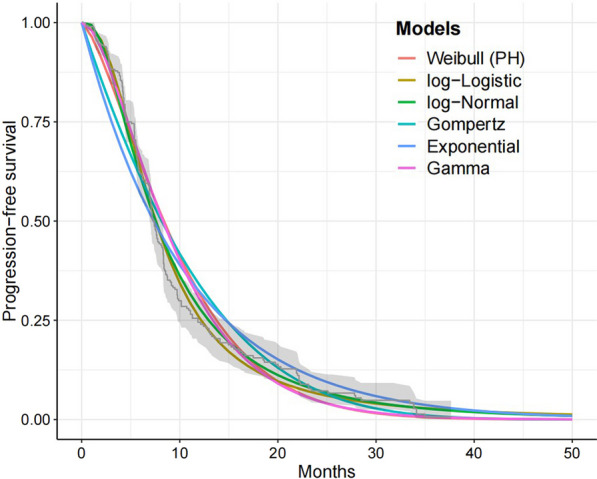
PFS(C) derived from the model simulation

**Fig. 14:**
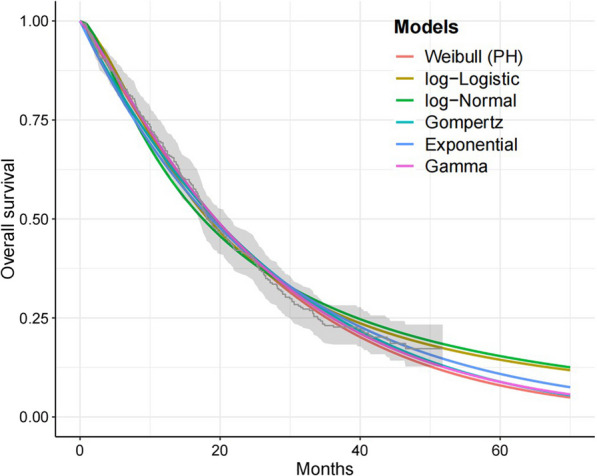
OS(NI-A) derived from the model simulation

**Fig. 15:**
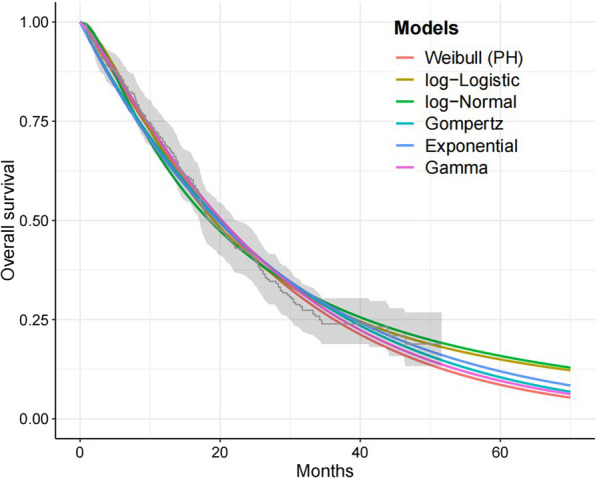
OS(NI-E) derived from the model simulation

**Fig. 16:**
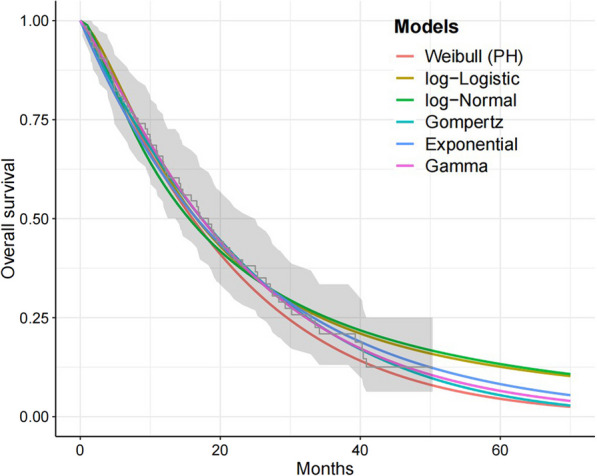
OS(NI-N) derived from the model simulation

**Fig. 17:**
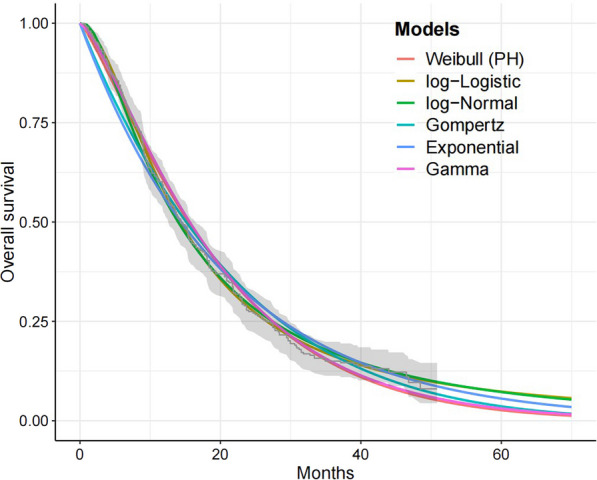
OS(C-A) derived from the model simulation

**Fig. 18:**
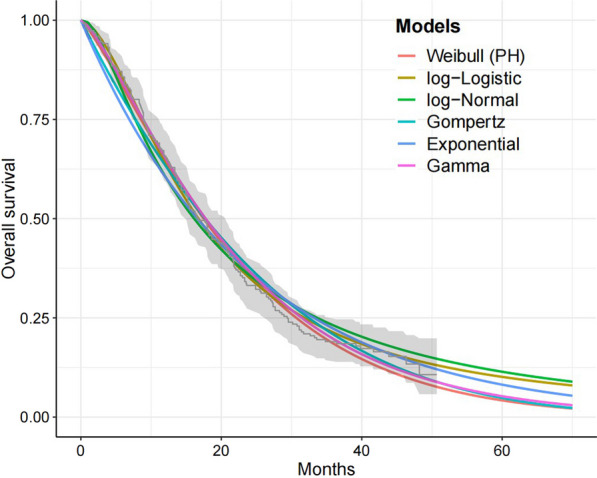
OS(C-E) derived from the model simulation

**Fig. 19:**
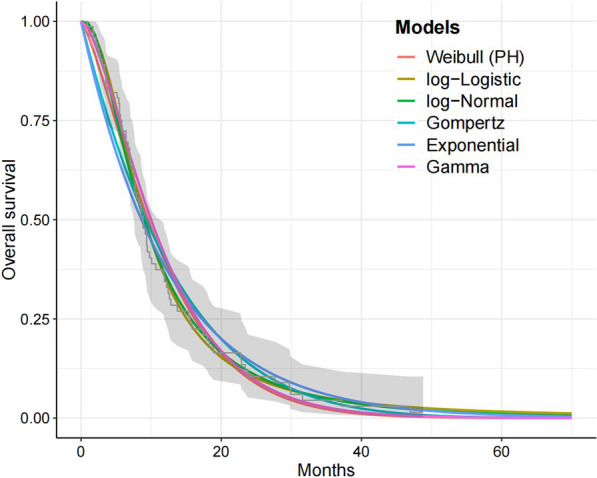
OS(C-N) derived from the model simulation

The Monte Carlo scatter plots of the probabilistic sensitivity analysis were shown in Figs. 5, 6 and 7. All the scatter points of the three populations were above the WTP threshold ($33,819), indicating that when the WTP is equal to 3 times the GDP per capita in China, the possibility of the NI scheme being more economical is 0. The cost-effectiveness acceptability curves for three different populations (Figs. 8, 9, 10) showed that as the WTP threshold increased, the probability that the NI option was more economical increased. However, when the WTP threshold was $33,819, the probability that NI was more economical than C was 0.

## Discussion

Compared with traditional chemotherapy, immunotherapy can bring more survival benefits to MPM patients, and the adverse events are also within an acceptable range. However, due to the high price of biologics, it is necessary to further evaluate the cost-effectiveness of the two regimens to judge whether NI regimen has economic advantages [10]. This study is the first to analyze the cost-effectiveness of the NI regimen in the treatment of MPM by constructing a Markov partition survival model. Although the CheckMate 743 trial showed a better survival benefit in the NI group, the results of this analysis show that NI is not an economically advantageous alternative to regimen C in the first-line treatment of patients with unresectable MPM from the perspective of the Chinese healthcare system. Among all randomized patients, the NI group had 0.10 QALYs more than the C group, with an incremental cost of $38,023 and an ICER of $375,656. In the epithelioid histological population, the NI group had 0.12 QALYs more than the C group, with an incremental cost of $38,002 and an ICER of $327,943. Among patients with non-epithelioid histology, the NI group had 0.33 QALYs more than the C group, with an incremental cost of $38,543 and an ICER of $115,495/QALY. In the three populations, the ICER values of the NI group were higher than the WTP threshold, that is, the NI scheme had no cost-effectiveness advantage. However, it is worth noting that the ICER of non-epithelioid histology population is about 1/3 of that of the epithelioid histology population. This may indicate that the NI regimen is more appropriate for the patients with non-epithelioid histology. When the WTP is about $115,000, the probability that the non-epithelioid histology population can afford it is 50%, while when the WTP is about $136,000, the probability increases to 90%.

The models constructed in this study all considered the influence of different second-line drugs on the analysis results. According to the MPM guidelines published by the NCCN [12], we assumed that patients in the NI group received pemetrexed plus cisplatin or carboplatin after the first progression, where the probability range of cisplatin and carboplatin was equal (baseline value = 0.5, range = 0–1). Group C received either immunotherapy or chemotherapy after the first progression, also assuming an equal range of probability (baseline value = 0.5, range = 0–1). Nivolumab monotherapy or combined ipilimumab was used for immunotherapy, and the range of probability of monotherapy or combination therapy was equal (baseline value = 0.5, range = 0–1). Chemotherapy was performed with vinorelbine or gemcitabine monotherapy, again assuming the same probability range for both (baseline value = 0.5, range = 0–1). We assumed that the first-line treatment time in both groups was equal to the median medication time in the CheckMate 743 study. However, NCCN guidelines [12], Chinese Guidelines for the Clinical Diagnosis and Treatment of Malignant Pleural Mesothelioma (2021 edition) [26] and related drug instructions do not clearly indicate the use cycle of nivolumab combined with or without ipilimumab, vinorelbine and gemcitabine in second-line treatment of unresectable MPM patients. Therefore, the duration of second-line treatment was determined according to the relevant references in the NCCN guidelines. Through one way sensitivity analysis, it was found that the price of pemetrexed had the greatest effect on the results in the three populations. And found that when the price of pemetrexed increased, the ICER value of the NI group compared with the C group gradually decreased. In the non-epithelioid histological population, the NI regimen had a cost-effectiveness advantage when the price of pemetrexed was greater than $5.41/mg. This paper considers that this may be related to the higher probability of first-line use of pemetrexed in group C than that of second-line use of pemetrexed in group NI. It is worth noting that the floating range of drug price was determined according to the bidding price in the past 5 years, and pemetrexed has been centrally purchased in many provinces in recent years, which has led to a significant drop in its price. Therefore, this study believes that it is difficult to see a situation where the price of pemetrexed is higher than $5.41/mg again. That is to say, this study believes that the NI group no longer has the conditions for cost-effective advantages due to the reform of the drug procurement policy.

This study still has some limitations. First, although the CheckMate743 trial provided OS curves for three different populations, it did not provide PFS curves for different populations. Therefore, for three different populations (all randomized patients, patients with epithelioid histology, and patients with non-epithelioid histology), their respective OS curves were used in this study. However, PFS curve of all randomized population was used to fit all survival partition models. Secondly, in order to simplify the model, this study only included adverse drug events with a large difference in incidence, and did not consider all adverse events and the utility value and treatment cost of AEs were obtained from published literature rather than real world data, which may lead to some bias. However, the results of univariate sensitivity analysis showed that these parameters had little impact on the results. Third, utility value is a key parameter for pharmacoeconomic evaluation, but since there was no accurate utility score in the published MPM-related literature, the utility parameters in this study referred to the published utility parameters of non-small cell lung cancer [27]. Although the one-way sensitivity analysis showed that the utility values of PFS and PD statuses played a certain role in the outcome analysis, it was also found that the ICER value was always higher than the WTP threshold no matter how the utility values of PFS status or PD status changed within the preset range. Finally, because the incidence of adverse events and corresponding treatment costs were lower in both groups, these costs were not discounted and were calculated using the 2020 cost data.

## What is new and conclusion

In 2017, Zhan et al. [27] compared the economics of bevacizumab combined with pemetrexed and cisplatin versus pemetrexed plus cisplatin in the treatment of patients with unresectable MPM naive to chemotherapy from the perspective of Chinese payers. Studies have confirmed that bevacizumab combined with pemetrexed and cisplatin is not a cost-effective treatment option for MPM in China. The pharmacoeconomic evaluation of MPM is very lacking, and these studies have the problems of low quality and long past. This is the first study on the economic evaluation of the first-line treatment of unresectable MPM with nivolumab combined with ipilimumab from the perspective of the Chinese healthcare system, which has a reference role in future clinical medication guidance and drug policy formulation. However, there are certain limitations, such as the failure to fully assess the factors affecting the health-related quality of life of patients.

The pharmacoeconomic evaluation conducted in this study conforms to standard methodological procedures [28]. Despite some limitations, the obtained results have high reliability. In other words, immunotherapy had no economic advantage over conventional chemotherapy as first-line treatment for patients with unresectable MPM when $33,819 was used as the WTP threshold. Given its positive clinical value and extremely low incidence of MPM, an appropriate price discount, assistance programs and medical insurance should be considered to make nivolumab plus ipilimumab more affordable for this rare patient population.

## Data Availability

All datasets for this study are included in the article as well as in the supplementary material.

## Appendix 1

See Table 3.

**Table 3:** Comparison of survival models

	AIC		BIC	
	Nivolumab plus ipilimumab	Pemetrexed plus platinum	Nivolumab plus ipilimumab	Pemetrexed plus platinum
OS-A				
WeibullPH	1985.716	1968.351	1993.143	1975.772
Log-logistic	1986.896	**1954.552**	1994.323	**1961.972**
Log-normal	1996.288	1958.015	2003.715	1965.436
Gompertz	1989.239	1980.054	1996.666	1987.474
Exponential	1988.198	1980.072	**1991.912**	1983.782
Gamma	**1984.816**	1963.240	1992.244	1970.661
OS-E				
WeibullPH	1477.448	1473.175	1484.315	1480.016
Log-logistic	**1474.177**	**1470.475**	**1481.044**	**1477.316**
Log-normal	1478.411	1486.525	1485.278	1493.366
Gompertz	1480.764	1480.351	1487.631	1487.192
Exponential	1479.087	1481.437	1482.520	1484.858
Gamma	1476.274	1471.601	1483.141	1478.442
OS-N				
WeibullPH	496.9260	475.9146	501.5341	480.5761
Log-logistic	499.4671	**468.2698**	504.0752	**472.9313**
Log-normal	502.2192	468.3080	506.8273	472.9695
Gompertz	497.4668	482.3946	502.0749	487.0561
Exponential	**496.1016**	481.7369	**498.4057**	484.0676
Gamma	496.8764	472.4620	501.4845	477.1235
PFS-A				
WeibullPH	1583.631	1448.885	1591.059	1456.306
Log-logistic	1536.415	**1419.132**	1543.842	**1426.553**
Log-normal	**1529.415**	1429.720	**1536.842**	1437.141
Gompertz	1549.582	1479.176	1557.010	1486.597
Exponential	1590.696	1488.348	1594.409	1492.058
Gamma	1590.134	1435.329	1597.561	1442.750

Bold standed for minimum value for each parameter (selected value)

*AIC* Akaike information criterion, *BIC* Bayesian Information Criterion, *OS* Overall survival, *PFS* Progression-free survival, *A* Randomized patients, *E* Patients with epithelioid histology, *N* Patients with non-epithelioid histology

## Appendix 2

See Figs. 11, 12, 13, 14, 15, 16, 17, 18 and 19.
